# Commentary: Cardiothoracic surgery and COVID: Must we coexist?

**DOI:** 10.1016/j.xjon.2020.11.003

**Published:** 2020-11-16

**Authors:** William Weir, Leah Schoel, Gorav Ailawadi

**Affiliations:** Department of Cardiac Surgery, University of Michigan Medical School, Ann Arbor, Mich

**Figure:**
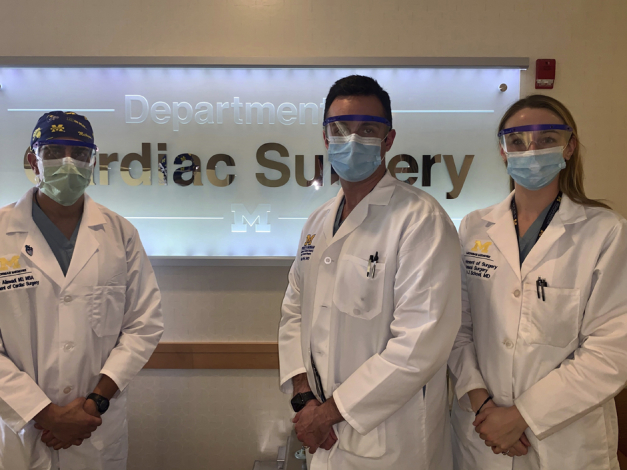
(*Left* to *right*) Gorav Ailawadi, MD, MBA, William Weir, MD, and Leah Schoel, MD

**Central Message**

Rigorous preoperative COVID testing does not compromise care and is imperative to team safety.

See Article page 107 in the December 2020 issue.

In a timely report in this issue of *JTCVS*, Balmforth and colleagues^1^ outline their single-institution protocol for preoperative screening at a large urban center in the United Kingdom. During the height of the COVID-19 pandemic, the authors report their experience with testing 76 cardiac surgery patients and 76 thoracic surgery patients before clearance for the operating room (OR). Of the implemented measures, including 2 separate nasal swabs, computed tomography scans, serum lactate dehydrogenase level, and total lymphocyte count, nasal swabs proved the most statistically reliable.

The STS Covid-19 Task Force proposed 3 central principles to guide operations during the viral pandemic: protecting the patient, protecting the cardiothoracic surgery team, and protecting the institution.^2^ Balmforth and colleagues clearly take the correct “patient-first” approach aimed at protecting patients from the reported 40% operative mortality when COVID-positive.^1^ We would argue that the next most crucial focus should be on protecting the highly specialized cardiothoracic surgery team. Yet without seeing data on infection rates among OR staff and postoperative care protocols, both of which are vital to any discussion about a COVID-19–free environment, the experience of Balmforth and colleagues is not complete. Cardiothoracic surgeons are tasked with leading the operative team, which includes protecting them from unnecessary risk. In many hospitals, illness of 1 or 2 members can bring an entire service offline. Thus, the only way to safeguard the patient, the OR team, and society as a whole is to institute rigorous preoperative protocols to keep the OR a COVID-free environment.

A rigorous screening protocol, as Balmforth and colleagues have outlined, is the linchpin of creating a COVID-free OR. Yet the screening protocol is only as good as the testing available. In an ideal world, to alleviate a false-negative rate of up to 29%, we advise that cardiothoracic surgery programs require 2 negative nasal swabs, one of which should be analyzed by reverse-transcription polymerase chain reaction.^3^^,^^4^ A single negative antigen test is not currently sufficiently reliable to safely proceed to surgery. Should the patient be intubated, we follow Balmforth and colleagues in recommending a deep tracheal aspirate.^1^

An often-underappreciated side of rigorous screening protocols is the decision to delay operation. In Balmforth and colleagues' cohort, 140 of 156 screened patients proceeded to surgery, which had a combined 5% operative mortality among cardiac and thoracic patients. Of those tested, 18 patients were positive for COVID, 13 of whom had their operations delayed. All patients who were delayed later underwent successful surgery, suggesting that in stable patients, waiting is not detrimental, and perhaps primary institutional focus should shift toward protecting the OR and hospital staff.

In short, surgical outcomes in COVID-positive patients are suboptimal and put staff at risk. Thus, outside of emergent clinical situations, cardiothoracic operations must be postponed in COVID-positive patients. Thus, COVID and cardiac surgery should not coexist unless in the most dire presentations. In the interim, we strongly advocate for aggressive patient testing, adequate personal protective equipment, and rapid isolation in positive patients to provide a safe, COVID-free OR environment.
